# Insights from the redefinition of *Helicobacter pylori* lipopolysaccharide O-antigen and core-oligosaccharide domains

**DOI:** 10.15698/mic2017.05.574

**Published:** 2017-04-25

**Authors:** Hong Li, Tiandi Yang, Tingting Liao, Aleksandra W. Debowski, Hans-Olof Nilsson, Stuart M. Haslam, Anne Dell, Keith A. Stubbs, Barry J. Marshall, Mohammed Benghezal

**Affiliations:** 1 West China Marshall Research Center for Infectious Diseases, Center of Infectious Diseases, Division of Infectious Diseases, State Key Laboratory of Biotherapy, West China Hospital of Sichuan University, Chengdu 610041, China.; 2 Helicobacter pylori Research Laboratory, School of Pathology & Laboratory Medicine, Marshall Centre for Infectious Disease Research and Training, University of Western Australia, Nedlands, Australia.; 3 Department of Life Sciences, Imperial College London, South Kensington Campus, London, SW7 2AZ, United Kingdom.; 4 School of Chemistry and Biochemistry, University of Western Australia, Crawley, Australia.; 5 Swiss Vitamin Institute, Route de la Corniche 1, CH-1066 Epalinges, Switzerland.

**Keywords:** Helicobacter pylori, lipopolysaccharide structure, persistence, therapy, antibiotic adjuvant

## Abstract

*H. pylori* is a Gram-negative extracellular bacterium, first discovered by the Australian physicians Barry Marshall and Robin Warren in 1982, that colonises the human stomach mucosa. It is the leading cause of peptic ulcer and commonly infects humans worldwide with prevalence as high as 90% in some countries. *H. pylori* infection usually results in asymptomatic chronic gastritis, however 10-15% of cases develop duodenal or gastric ulcers and 1-3% develop stomach cancer. Infection is generally acquired during childhood and persists for life in the absence of antibiotic treatment. *H. pylori* has had a long period of co-evolution with humans, going back to human migration out of Africa. This prolonged relationship is likely to have shaped the overall host-pathogen interactions and repertoire of virulence strategies which *H. pylori* employs to establish robust colonisation, escape immune responses and persist in the gastric niche. In this regard, *H. pylori* lipopolysaccharide (LPS) is a key surface determinant in establishing colonisation and persistence via host mimicry and resistance to cationic antimicrobial peptides. Thus, elucidation of the *H. pylori* LPS structure and corresponding biosynthetic pathway represents an important step towards better understanding of *H. pylori* pathogenesis and the development of novel therapeutic interventions.

Earlier studies of the *H. pylori* LPS structure proposed that the core-oligosaccharide domain encompasses an inner core and outer core and inferred the attachment site of the O-antigen composed of Lewis antigens only, as depicted in Figure 1A. Our structural analyses of LPS from both wild-type strain and isogenic O-antigen ligase mutant revealed that the core-oligosaccharide is a short hexa-saccharide comprised of Glc-Gal-DD-Hep-LD-Hep-LD-Hep-KDO, indicating that the trisaccharide (GlcNAc-Fuc-DD-Hep) termed as Trio, the glucan and the heptan structure, previously assigned as the outer core, should all be redefined as part of the O-antigen (Figure 1B). According to our redefinition of the *H. pylori* LPS O-antigen domain, the GlcNAc residue of the Trio is transferred by WecA that initiates the biosynthesis of the long *H. pylori* LPS O-antigen (Figure 1B). The linear architecture of the heptan-glucan-Trio was observed in different strains. This raises an interesting question about the role of this general feature of *H. pylori* LPS in pathogenesis. Furthermore, the conservation of the Trio moiety of the newly defined O-antigen contrasts with the variability of the distal domains of O-antigen present in different strains. On the one hand, the variability of the O-antigen beyond the conserved Trio may enable *H. pylori* to adapt to specific human hosts - of different geo-graphical regions for example - via host mimicry and the presence of Lewis antigens or possibly of other determinants as reported in Danish strains. On the other hand, the conserved core and Trio may reflect evolutionary adaptation to shared mammalian traits such as the innate immune system, enabling the survival and persistence of *H. pylori *within the gastric mucosa.

**Figure 1:**
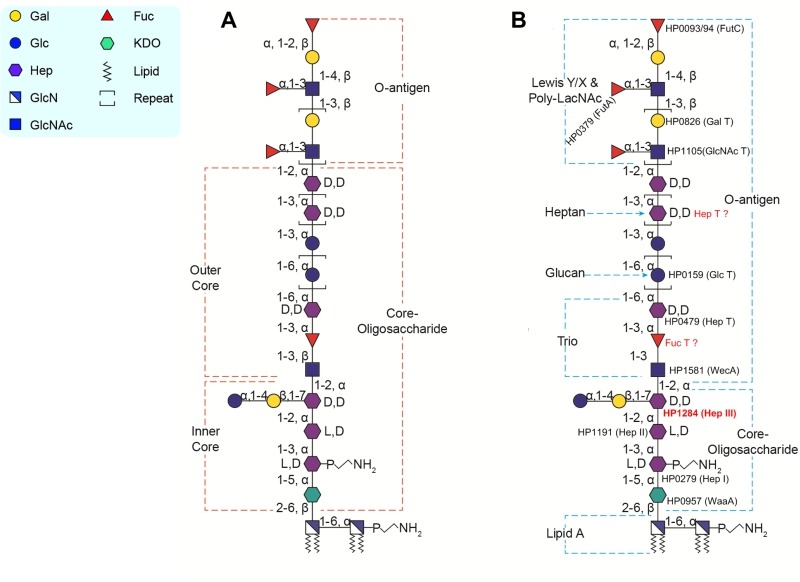
Figure 1: Previously proposed and the redefined structures of *H. pylori* LPS from strain 26695. **A)** Previously in *H. pylori* strain 26695, the LPS O-antigen domain was proposed to contain the Lewis antigen only, the LPS core-oligosaccharide domain was divided into an inner core and outer core. The inner core is a hexa-saccharide comprised of Glc-Gal-DD-Hep-LD-Hep-LD-Hep-KDO, the outer core is comprised of the Trio, the heptan and glucan. **B)** Based on our recent study, the LPS structure in *H. pylori* strain 26695 is redefined: the O-antigen encompasses more than the Lewis antigen, but also the previously defined outer core structure (the Trio, the glucan and the heptan), whereas the core-oligosaccharide only contains the short hexa-saccharide which was previously assigned as the inner core. Figure reproduced from Li *et al*. 2017 (doi: 10.1371/journal.ppat.1006280) under the Creative Commons CC BY 4.0 license.

Not all glycosyltransferases of the *H. pylori* LPS biosynthetic pathway have been identified. A potential reason for this lies in that *H. pylori* LPS genes are scattered over the whole *H. pylori* genome instead of being organised in an operon as it is usually the case in other Gram-negative bacteria. Prior to our study, glycosyltransferases responsible for transferring the Hep III, the GlcNAc and Fuc residues of the Trio, and the heptan were unknown. Using a targeted approach, our group identified the conserved putative heptosyltransferase HP1284 as required for the transfer of the Hep III residue to the core-oligosaccharide. Taking into account previously characterised LPS biosynthetic genes, known glycosyltransferases were assigned onto the complete structure of *H. pylori* including HP1284 (Figure 2). Currently, a systematic genetic deletion campaign for all *H. pylori *glycosyltransferases has been initiated in our laboratory for the discovery of the missing genes of *H. pylori* LPS biosynthetic pathway (Figure 2).

**Figure 2:**
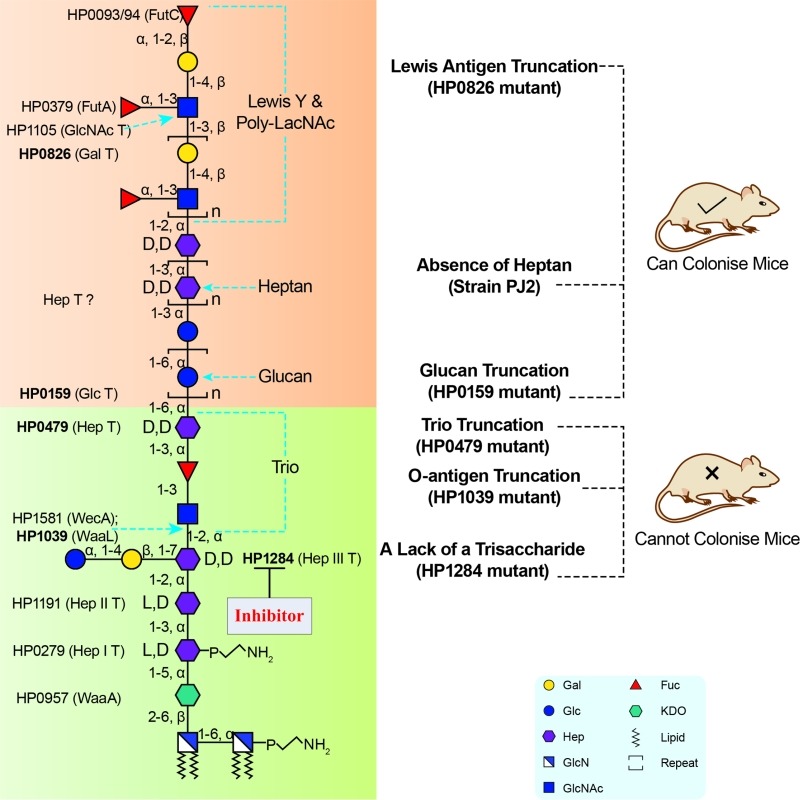
Figure 2: Inhibitor targeting the *H. pylori* LPS biosynthesis pathway. LPS structure up to the Trio is conserved among *H. pylori* strains and required for colonisation. Thus, corresponding LPS biosynthetic enzymes involved in the assembly of the LPS conserved domains, such as HP1284, represent attractive virulence targets for the design of novel therapeutic agents for managing persistent *H. pylori* infection. Figure reproduced from Li *et al*. 2017 (doi: 10.1371/journal.ppat.1006280) under the Creative Commons CC BY 4.0 license.

Our study also demonstrated that mutants deficient in either O-antigen ligase WaaL or HP1284 were less resistant to polymyxin B and unable to colonise the mouse gastric mucosa. Mapping LPS mutagenesis and colonisation data of previous studies onto our redefined *H. pylori* LPS structure revealed that the conserved Trio and the short core of *H. pylori* LPS are essential for colonisation, suggesting that enzymes involved in the assembly of the conserved core structure, such as HP1284 and WaaL could be attractive virulence targets for the design of novel therapeutic agents for managing persistent *H. pylori* infection (Figure 2).

Inhibitors of bacterial virulence factors which interfere with bacterial pathogenesis mechanisms have been proposed as an alternative to antibiotics and may bring a solution to the increasing problem of antibiotic resistance. LPS contributes to the structural integrity of the bacterial outer membrane and acts as a shield against external chemical and immunological attacks, including antibiotics and cationic antimicrobial peptides of the innate immune system. In *E. coli*, micromolar inhibitors of the glycosyltransferase WaaC (responsible for transferring the first Hep unit to the lipid A core of the LPS) and inhibitors of the HldA and HldE enzymes (involved in the Hep biosynthetic pathway) have been identified by virtual screening and tested successfully *in vitro*, suggesting that inhibitors targeting the LPS biosynthetic pathway have a therapeutic potential. In the case of *H. pylori* LPS, the O-antigen mimics the host’s Lewis blood group antigens and binds to the DC-SIGN receptor of dendritic cells inducing tolerance rather than immunity. Consequently, inhibition of LPS biosynthesis in *H. pylori* may have a dual mode of action by decreasing both resistance to cationic peptides and immune tolerance, facilitating bacterial clearance by the host.

The standard first-line therapy to eradicate *H. pylori* infection, once *H. pylori* are detected in symptomatic patients, is a one week triple therapy consisting of the antibiotics amoxicillin and clarithromycin, with a proton pump inhibitor such as omeprazole. Major issues of concern worldwide are the unsatisfactory eradication rates of less than 80% and the emergence of antibiotic resistant clinical strains which do not respond to these current therapies and which will in turn lead to even lower eradication rates. Thus, anti-virulence inhibitors targeting *H. pylori* LPS have the potential to be used as antibiotic adjuvants in a synergistic manner to improve the efficacy of triple antibiotic therapy.

In summary, the elucidation of *H. pylori*’s complete LPS structure has led to the redefinition of its core and O-antigen domains. This new knowledge will drive future studies to investigate precisely the role of each domain in the pathogenesis of this human gastric pathogen. Furthermore, the discovery of the remaining glycosyltransferases of the *H. pylori* LPS pathway will enable genome-wide comparative bioinformatic studies to better understand how *H. pylori* can remodel the major component of outer membrane to adapt to and persist in the gastric niche. In view of the triple antibiotic therapies required to eradicate *H. pylori* infection, inhibition of LPS biosynthesis could potentiate currently used antibiotics by permeabilising the outer membrane, slowing the emergence of resistance, interfering with host mimicry and achieving much needed higher eradication rates.

